# The analysis and reporting of multiple outcomes in mental health trials: a methodological systematic review

**DOI:** 10.1186/s12874-024-02451-8

**Published:** 2024-12-21

**Authors:** Dominic Stringer, Mollie Payne, Ben Carter, Richard Emsley

**Affiliations:** Department of Biostatistics and Health Informatics, Institute of Psychiatry, Psychology and Neuroscience, Kings College London, London, UK

**Keywords:** Clinical trials, Mental health, Outcomes, Complex interventions, Multiplicity, CONSORT

## Abstract

**Background:**

The choice of a single primary outcome in randomised trials can be difficult, especially in mental health where interventions may be complex and target several outcomes simultaneously. We carried out a systematic review to assess the quality of the analysis and reporting of multiple outcomes in mental health RCTs, comparing approaches with current CONSORT and other regulatory guidance.

**Methods:**

The review included all late-stage mental health trials published between 1st January 2019 to 31st December 2020 in 9 leading medical and mental health journals. Pilot and feasibility trials, non-randomised trials, and early phase trials were excluded. The total number of primary, secondary and other outcomes was recorded, as was any strategy used to incorporate multiple primary outcomes in the primary analysis.

**Results:**

There were 147 included mental health trials. Most trials (101/147) followed CONSORT guidance by specifying a single primary outcome with other outcomes defined as secondary and analysed in separate statistical analyses, although a minority (10/147) did not specify any outcomes as primary. Where multiple primary outcomes were specified (33/147), most (26/33) did not correct for multiplicity, contradicting regulatory guidance. The median number of clinical outcomes reported across studies was 8 (IQR 5–11 ).

**Conclusions:**

Most trials are correctly following CONSORT guidance. However, there was little consideration given to multiplicity or correlation between outcomes even where multiple primary outcomes were stated. Trials should correct for multiplicity when multiple primary outcomes are specified or describe some other strategy to address the multiplicity. Overall, very few mental health trials are taking advantage of multiple outcome strategies in the primary analysis, especially more complex strategies such as multivariate modelling. More work is required to show these exist, aid interpretation, increase efficiency and are easily implemented.

**Registration:**

Our systematic review protocol was registered with the International Prospective Register of Systematic Reviews (PROSPERO) on 11th January 2023 (CRD42023382274).

**Supplementary Information:**

The online version contains supplementary material available at 10.1186/s12874-024-02451-8.

## Background

An important aspect of conducting a clinical trial is the selection, analysis and reporting of outcomes to address the trial’s objectives and evaluate the intervention. CONSORT 2010 guidance [1] recommends explicitly pre-specifying a single primary outcome. The primary analysis, evaluating the primary outcome, usually aims to provide a clear and definitive answer about the efficacy or effectiveness of the intervention being studied. The required sample size is then typically justified based on achieving a specified power to detect the smallest clinically important difference on the primary outcome. Additional outcomes may be defined as secondary and used to support the primary results or address additional objectives but are customarily intended to be interpreted as less confirmatory.

The selection of a single primary outcome can be challenging, particularly in trials of complex interventions, which are common in mental health. Complex interventions may have multifaceted components [2], targeting different aspects of the mental health condition. An example is cognitive remediation therapy for psychosis [3], which is a therapy that involves 4 effective elements “cognitive exercise, developing problem-solving strategies, an active therapist, and facilitating transfer to real-world functioning” [3, 4] through computer exercises and discussion with a therapist. This intervention targets both cognitive abilities and functioning and these outcomes would be measured using different constructs.

Mental health trials often need to rely on subjective self-reported or clinician rated measures such as psychological questionnaires rather than direct measurement (alongside “hard” outcomes such as mortality which are still important but may occur less frequent in mental health conditions) There is currently little harmonisation of core outcomes in mental health [5] as seen in other disciplines through initiatives such as COMET [6] Perhaps as a result of this, mental health trials often report more outcomes than in other clinical areas [7]. These outcomes can overlap, especially where they represent transdiagnostic processes (for example low mood may be underpinned by separate measures of depression, anxiety and sleep).

DDecisions by stakeholders, regulators and other decision makers, such as the NICE in the UK, on the implementation of an intervention do not depend on a single outcome. Harms, cost-effectiveness, and process outcomes such as acceptability of the intervention, are examples of other types of outcomes that a trial may wish to include for decision making regarding the intervention. In this paper we focus purely on the multiplicity of clinical outcomes (outcomes that reflect how the patient feels or functions).

Multiple clinical outcomes may be needed where the views of stakeholders differ, where the intervention may be posited to affect different aspects of the disease simultaneously, or where there are multiple imperfect measures of the underlying target aspect of the disease. Designating these as secondary outcomes may not always be sufficient as secondary outcomes are intended to be interpreted as more exploratory. Multiple primary outcomes can be defined but current CONSORT 2010 guidance warns against this; without multiplicity correction or another strategy, this can lead to an increased chance of a type I error [8] allowing authors to erroneously increase the chance of claiming the trial to be successful based on 1 of the multiple primary outcomes being found to be “significant”. A recent extension to CONSORT, CONSORT-Outcomes 2022 [9] extends these recommendations; prompting authors to clearly describe the multiplicity issues when using multiple primary outcomes and any methods used to account for them.

In a previous systematic review by Vickerstaff et al. [10], they examined whether multiple primary outcomes were assessed appropriately in mental health and neuroscience trials from 2011 to 2014, published in high impact journals. The review focused on whether they used an appropriate multiplicity correction for multiple primary outcomes. This current review provides a contemporaneous update to Vickerstaff et al. [10] but with a greater focus on whether mental health trials are utilising any “multiple outcome strategies” for the primary analysis. Here we are defining “multiple outcome strategies” as a broad term for analysis or reporting methods that account for the multiplicity, relatedness or correlation of multiple trial outcomes. In addition to correction for multiplicity, examples of other multiple outcome strategies would be composite outcomes, global hypothesis testing and multivariate modelling. These approaches represent alternative ways to use multiple outcomes in the primary analysis to aid interpretation. One example is the win ratio, a composite/global test that has been used particularly in cardiovascular trials [11].

The objective of this systematic review is to assess the current state of analysis and reporting of multiple outcomes in randomised controlled trials in mental health. The key aims are to assess the following:

I. Whether mental health trials are currently following CONSORT reporting guidelines and other regulatory guidance [9, 12–14], specifically with regards to defining a clear single primary outcome or adequately describing/accounting for the multiplicity of multiple primary outcomes.
II. Whether mental health trials are currently utilising multiple outcome strategies to address primary trial objectives or increase power.

## Methods

This study was reported following the preferred reporting items for systematic reviews and meta-analysis (PRISMA) guidance ( [15]), following a registered protocol on PROSPERO (CRD42023382274).

### Study selection and data sources

The review includes all randomised controlled trials of interventions for mental health in humans published between 1st January 2019 and 31st December 2020 in leading medical and mental health journals; *The British Journal Of Psychiatry*, *Lancet Psychiatry*, *Jama Psychiatry*, *Journal Of The American Academy Of Child And Adolescent Psychiatry*, *Psychiatry Research*, *Psychological Medicine*, *American Journal Of Psychiatry*, *The Lancet*, *BMJ* and *Nature*. These journals were chosen to represent the leading journals that mental health trials would likely be published in. A mental health trial was defined as a trial where the target population was participants with a mental health condition or were expected may develop a mental health condition.

Pilot and feasibility trials, non-randomised trials, and early phase (Phase 1 or Phase 2a) trials were excluded. Only primary trial results papers were considered; published secondary or interim analyses of trial data were not included. The 2019–2020 timeframe was chosen so the review might reflect recent practice but would not include trials affected by the unique challenges of conducting a trial during the COVID-19 global pandemic.

### Search strategy

The search was performed in the Ovid MEDLINE database (see supplementary material S7 for full search criteria). Two reviewers independently screened all titles and abstracts. Full texts were reviewed by both reviewers where eligibility was not clear from the abstract. Data was extracted onto a pre-piloted standardised data extraction form (DS) for all eligible articles; with a random 20% sample independently extracted by a second reviewer (MP) to check consistency. Disagreements were resolved by discussion or by a third reviewer (RE). Only information from the paper as published was used for the extraction, supplementary materials including protocols, trial registry data and statistical analysis plans were not used in order to represent current trial reporting and simplify data extraction.

Due to the nature of the review, no quality assessment tool was used.

### Data extraction

Characteristics of the included studies that were recorded were: sponsor; trial design; type of intervention (drug, complex intervention or other); phase; sample size; target population (assessed and categorised by the authors rather than directly extracted); mean age of randomised participants and percentage of participants who were female.

For each trial, the total number of clinical outcomes analysed and reported was counted, including number explicitly or implicitly defined as primary, secondary, exploratory or otherwise undefined. Adverse events, other than where clearly also defined as a clinical measure of efficacy or effectiveness, and other non-clinical outcomes were not counted. For the purpose of the review, outcomes that were recorded and analysed at repeated timepoints were counted only once, regardless of how they were reported.

### Outcomes

We recorded whether a single primary outcome was explicitly and clearly stated and if any strategy had been used to incorporate multiple outcomes in forming the primary analysis. We determined if any multiplicity correction had been made, either for multiple primary outcomes or for additional outcomes. We recorded whether the methods section justified why the authors did or did not make a multiplicity correction. For both composite and multicomponent primary outcomes (which were counted as single outcomes), we recorded whether components or sub-scales were also analysed separately.

Additional outcomes for the review examined whether there were secondary or supplementary analyses that made use of multiple outcomes in a single analysis. Data on whether multiple timepoints were incorporated in the primary analysis was also captured i.e., whether a pre-specified timepoint was stated (as recommended per CONSORT 2010 guidance), whether the repeated measures were analysed in a single model and whether individual effects were reported at each timepoint or if a summary measure was reported.

### Data synthesis and subgroup analysis

The total number of outcomes was summarised using medians and lower and upper quartiles. Frequencies and percentages were used to summarise categorical outcomes. Data was analysed in Stata 17. Studies were further split and summarised by the following subgroups:

i) Trials of complex interventions versus non-complex interventions (medications or other non-complex).
ii) Parallel group designs versus other trial designs (e.g., cluster randomised).
iii) Trials sponsored or funded by for-profit organisations versus non-for-profit organisations.

Functional magnetic resonance imaging (fMRI) trials are reported separately.

### Definition of multiple outcome strategy

For the purpose of this review, a multiple outcome strategy was defined as any analysis or reporting strategy that incorporated multiple outcomes in the primary analysis. Primarily, this could involve using multiplicity adjustment, composite outcomes, global test statistics or multivariate modelling. These are further described below:

Multiplicity adjustment involves correcting *p*-values for the number of outcomes/comparisons to preserve the overall type 1 error; multiple methods exist, the Bonferroni-based adjustment [16] being the most well-known. Other less conservative examples include the Sidak correction [17] and the Hommel/Simes multiple testing procedure [18].

A composite outcome is a single outcome defined as a combination of multiple separate components [19]. Composites are frequently event-based i.e., whether a participant has experienced one or more of the qualifying events. However, composites of continuous measures or other data types are possible; often combined linearly and/or including weights for the individual components.

Another approach is to test effects from different endpoints using a single global test statistic. As opposed to a composite outcome, in this approach, treatment effects may be estimated individually on the original endpoints, but the hypothesis testing is carried out using an overall test on some combined variate. Such tests may be parametric or non-parametric. Examples of global tests include O’Brien’s test [20], the Wei-Lachin test [21] and the Win ratio [22].

Multivariate modelling can be seen as an extension of global testing, where the relationship between multiple outcomes and the treatment is modelled simultaneously. This can allow for estimation of treatment effects on each outcome individually or combined, and also account for the correlation between outcomes to potentially increase statistical power, especially in presence of missing data. Multilevel models [23] and latent variable models [24] are two examples of proposed methods. The latent variable approach models the correlation between outcomes as a common latent factor. Multilevel models use random effects to model the multivariate hierarchical structure.

## Results

### Included articles

**Fig. 1:**
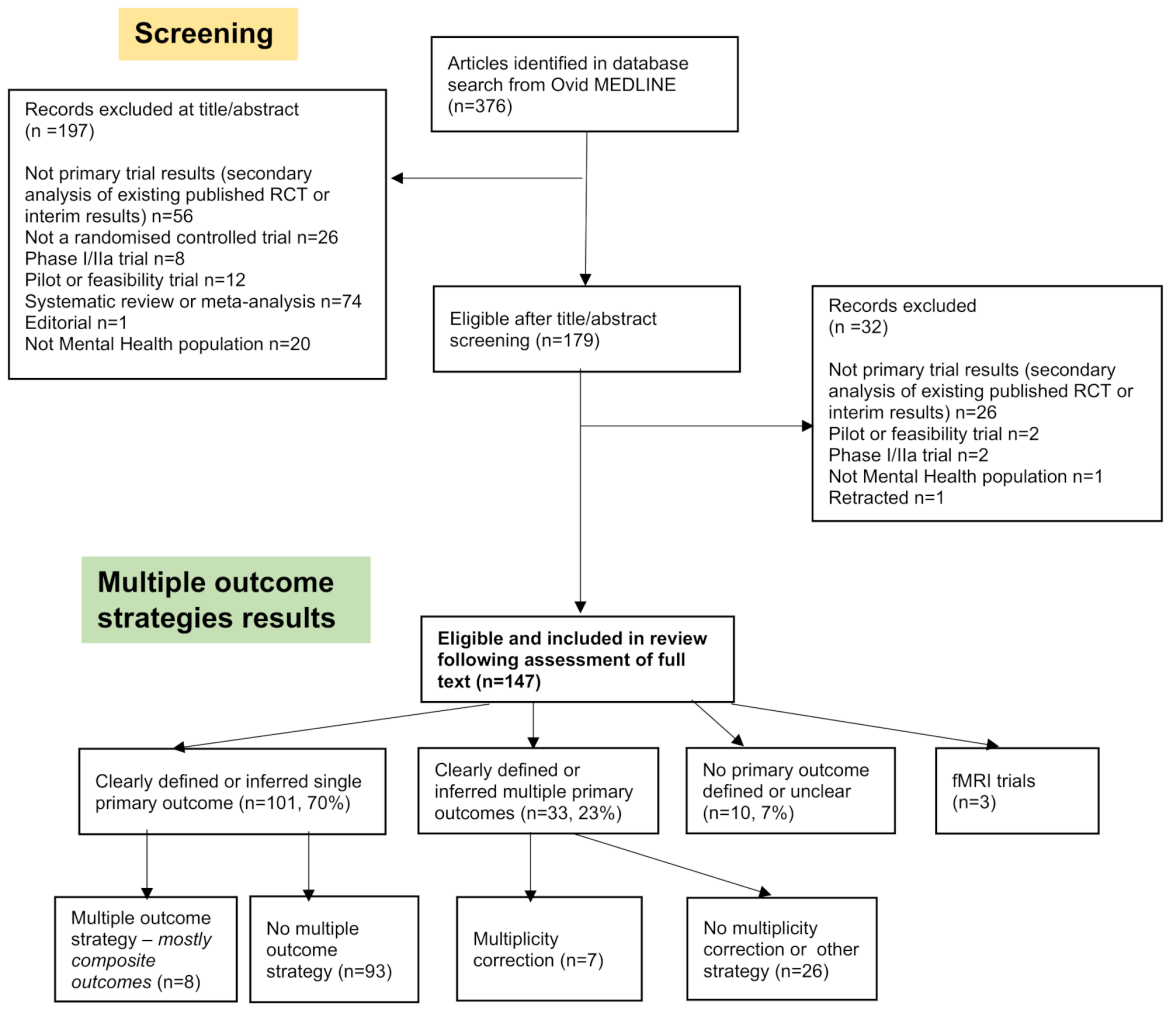
Included articles flow diagram (PRISMA Flowchart)

Of the 376 articles identified during the Ovid MEDLINE database search, 147 eligible trials were identified and included in the review. The PRISMA flowchart (Fig. 1) provides reasons for exclusions.

### Characteristics of the included studies

Table 1 describes the characteristics of the 147 included studies. Additional characteristics are described in the supplementary materials (Section S1).

**Table 1:** Characteristics of included trials

Characteristic	*n*	(%)
**Journal**		
JAMA psychiatry	38	(25.9)
Psychological medicine	31	(21.1)
The British journal of psychiatry : the journal of mental science	18	(12.2)
The American journal of psychiatry	17	(11.6)
Depression and anxiety	14	(9.5)
The Lancet Psychiatry	14	(9.5)
Journal of the American Academy of Child and Adolescent Psychiatry	11	(7.5)
The Lancet	3	(2.0)
The BMJ	1	(0.7)
Nature	0	(0)
**Year**		
2019	69	(46.9)
2020	78	(53.1)
**Sponsor**		
Academic or not-for-profit	132	(89.8)
Pharmaceutical or for-profit	15	(10.2)
**Trial Design**		
Parallel	120	(81.6)
Cluster randomised	16	(10.9)
Other^1^	7	(4.8)
Crossover	4	(2.7)
**Type of Intervention**		
Complex intervention	106	(72.1)
Drug	32	(21.8)
Device or other non-complex intervention	9	(6.1)
**Trial phase** ^**2**^		
IIb	26	(17.7)
IIb/III	48	(32.7)
III	70	(47.6)
IV	3	(2.0)
**Number of arms**		
2	121	(82.3)
3	19	(12.9)
4	5	(3.4)
>4	2	(1.4)
**Target population - Categorised**		
Depressive disorders	37	(25.2)
Schizophrenia spectrum and other psychotic disorders	20	(13.6)
Multiple categories/other	17	(11.6)
Trauma- and stressor-related disorders	16	(10.9)
Anxiety disorders	13	(8.8)
Substance-related and addictive disorders	10	(6.8)
Neurodevelopmental disorders	9	(6.1)
Bipolar and related disorders	5	(3.4)
Feeding and eating disorders	5	(3.4)
Sleep–wake disorders	3	(2.0)
Neurocognitive disorders	3	(2.0)
Obsessive-compulsive and related disorders	2	(1.4)
Somatic symptom and related disorders	2	(1.4)
Personality disorders	2	(1.4)
Disruptive, impulse-control, and conduct disorders	2	(1.4)
Sexual dysfunctions	1	(0.7)

^1^Of the 7 trials with other designs, these included 3 Factorial trials, 2 Randomised preference trials, 1 SMART (Sequential Multiple Assignment Randomized) trial and 1 stepped wedge cluster design

^2^For most trials, phase was not explicitly stated and was inferred based on the objectives stated, sample size and other design characteristics

Only four trials (2.7%) were reported from general medical journals; almost all were reported in specialist mental health journals (*n* = 143). The majority were academic (or not-for-profit) initiated (132, 90%), with fewer sponsored by pharmaceutical companies or other for profit entities (15, 10%). The majority of trials (106, 72%) evaluated a complex intervention, followed by a medicinal product (32, 22%) or other type of intervention (9, 6%). However, very few trials (8/106, 8%) stated explicitly that the intervention was complex, with most interventions inferred by the reviewers as complex as they appeared to have multiple interacting components. Phase of the trial was also mostly inferred (140, 95%), not unexpectedly as trial phase is based on a drug development framework [25] and most of the trials were of complex interventions. The majority of trials were assessed as Phase III (70,48%) or as Phase IIb and/or Phase III (48, 33%), although the latter category was used when it was unclear which of these phases applied. The identified trials cover a wide spectrum of different mental health conditions.

Most trials included were two arm (122, 83%) and parallel group (120 ,82%), with a small number cluster randomised (16,11%), crossover (4,3%), or having another design (7, 5%).

The time that the trials started recruitment spanned over a long period of time, from 2000 to 2019 (Supplementary table S1).

### Number of outcomes reported

The median total number of clinical outcomes reported was 8 (IQR: 5–11). 52 trials (36%) reported more than 10 separate clinical outcomes.

97 (67%) trials clearly stated a single primary outcome as per CONSORT 2010 guidance. An additional 4 trials appeared to have a single primary outcome, but this was inferred rather than clearly stated.

29 (20%) trials clearly stated that they had multiple primary outcomes. Another 4 were inferred as having multiple primary outcomes. The median number of primary outcomes in these trials was 2 (IQR: 2–3) with a mean of 2.9 primary outcomes. The highest number of primary outcomes was 13, in this case the trial specified several domains as primary outcomes which each incorporated several separate measures, namely parent and adolescent ratings of sleepiness, ADHD symptoms and oppositional behaviours. Many of the other trials with a large number of primary outcomes also specified one or more domains that actually translated into several separate measures.

10 (6.9%) trials did not clearly define any outcome(s) as the primary outcome, and this was not able to be inferred as for each of these trials, none of the outcomes were defined.

125 (86%) trials defined secondary outcomes explicitly, the median number of secondary outcomes reported was 4 (IQR: 1–8). Very few trials (11, 8%) described any outcomes as exploratory outcomes. 40 (28%) trials reported additional outcomes that were not defined as primary, secondary or exploratory or 51 (35%) including the 11 trials described above which did not define any of the outcomes.

**Multiple outcome strategies (for primary analysis)**.

Few trials reported (*n* = 15, 10%) using any strategy to analyse or report multiple outcomes as part of the primary analysis. Of these, 8 trials reported (or it was inferred) that the primary measure was a composite endpoint with 5 trials using an unweighted composite of events (composites were counted as a single outcome so are part of the *n* = 100 trials with a single primary outcome as above). For example, Daly et al. [26] used relapse as the primary outcome, which was defined as a Montgomery–Åsberg Depression Rating Scale (MADRS) total score of 22 or higher or hospitalisation for worsening depression or suicide attempt. Two trials used composites of continuous measures without reporting weights (and therefore presumably used a linear combination with equal weights). One trial (Haight et al. [27]) used a composite percentage; abstinence from opioid use defined as “the percentage of each participant’s negative urine samples and self-reports of illicit opioid use among 20 weekly opioid use assessments”. Implicitly this was also unweighted. Of the 8 trials that reported a composite, 5 also analysed the individual components separately.

None of the trials identified in the review reported using global statistical tests, multivariate modelling, or any approach that might account for the correlation between outcomes, to incorporate multiple outcomes in the primary analysis.

### Multiple primary outcomes

7/29 (24%) of trials which clearly stated multiple primary outcomes used a multiplicity correction to account for these, which we counted as a multiple outcome strategy for the purpose of this reviewOf these, 2 trials used a Bonferroni correction, while one used a Bonferroni correction only within domain (7 primary outcomes were defined, split into 3 domains). 3 trials did not specify a named correction method but stated that they would use a lower alpha for each outcome to correct for multiple testing for the primary outcomes. One of these trials justified this lower alpha based on the correlation among the primary outcomes. One trial specified that they used a group sequential test procedure described by Cui et al. [28] although this appeared to be to adjust only for the interim analysis rather than the multiple primary outcomes.

The other 23/29 (79%) trials that stated multiple primary outcomes made no multiplicity correction. None of the additional 4 trials that we inferred had multiple primary outcomes used multiplicity correction. In the previous review by Vickerstaff et al. [10], co-primary outcomes were differentiated from multiple primary outcomes, with co-primaries defined as needing to show an effect on all co-primary outcomes, precluding the need for multiplicity correction. 3/29 trials reported the primary outcomes explicitly as “co-primary” (and of the other 26/29, we could not infer that any considered the primary outcomes as “co-primary”) although 2 of these trials did still correct for multiple testing.

### Additional strategies for other outcomes

Of the trials with a single primary outcome, 18/101 (18%) reported using a multiplicity correction for secondary or additional outcomes. A small number of trials (5) used strategies that incorporated several secondary or other undefined outcomes as part of a single analysis. 4 of these used unweighted composites (2 continuous, 2 event-based). One trial used principal component analysis to create overarching measures of behaviour from child and parent rated scales.

### Multicomponent outcomes

A large proportion of identified trials (68%) used a multicomponent measure as the primary outcome, these were counted as a single outcome. Multicomponent was loosely defined for the purpose of this review as any outcome that is commonly treated as a single outcome but arises from multiple questions or measures. Usually, this meant a validated psychometric measure, which may or may not have had additional subscales. 15 (15%) trials with a multicomponent primary outcome also reported and analysed individual components or subscales as separate outcomes.

### Longitudinal, using repeated measures

119 trials recorded the primary outcome measure at multiple follow up timepoints. 68/119 (57%) trials prespecified one of the timepoints as the primary contrast. 106/119 (89%) specified that they used a single model to analyse all of the timepoints, with 7 trials analysing the timepoints separately (and 6 trials not adequately describing the analysis to determine this). Of the 106 trials which used a single model, 77 (73%) reported treatment effects at each of the timepoints, with 27/77 additionally reporting a summary measure. 28/106 (26%) trials reported only a summary measure. For 1 trial it was unclear. The summary measure reported was most often a treatment x time interaction effect or equivalently the difference in slopes over time.

### Complex interventions

The number of trials that explicitly stated the primary outcome(s) was similar for trials of complex interventions compared to non-complex interventions, with a slightly higher proportion of complex intervention trials (34/103, 33% compared to 9/41, 22%) stating multiple primary outcomes. The median number of clinical outcomes was similar for trials of complex interventions compared to non-complex interventions.

### For profit vs. not for profit

There was only a small number of for-profit trials (15) captured in the review. There did appear to be a higher proportion of for-profit trials that clearly stated the primary outcome compared to not-for-profit trials, and a lower median number of outcomes reported in the for-profit trials. A larger proportion also used a multiple outcome strategy (5/15, 33%) compared to not for profit (10/129, 8%). Conversely, there was a lower percentage who reported multiple primary outcomes (2/15, 13% versus 41/129, 31%).

### Parallel versus Cluster randomised trials

As there were only a small number of crossover or other study designs, we compared parallel individually randomised trials against cluster randomised trials only. Cluster and parallel trials had similar rates of clearly stating the primary outcome(s), although a higher proportion of the cluster randomised trials reported multiple primary outcomes (7/16, 44% vs. 34/118, 29%). Similar rates of using a multiple outcomes strategy were observed across both types of study design.

### Functional magnetic resonance imaging (fMRI)

Three trials reported outcomes exclusively using fMRI data which we considered separately and are not counted in the reported numbers above. fMRI outcomes are multidimensional as they consider changes across multiple different brain regions and multivariate analysis is typically used. Of these trials, one clearly stated brain changes as the intended primary, in the other two trials this was unclear or not mentioned. 2 of the 3 fMRI trials reported using multiplicity correction.

## Discussion

### Recommendations

The low number of trials which used multiple outcome strategies imply such approaches may be underutilised. Of the few strategies used, these were mostly simplistic, involving multiplicity correction (which reduce efficiency) or composite outcomes. The trials which used composite outcomes did so in a straightforward way without weighting, and most did not additionally report effects on the components separately. Much guidance cautions against composite measures as composites can be driven by the least important component, so it is recommended that results on the individual components should be additionally reported separately [14] and weighting should be considered [29].

Most trials in the review did specify a single primary outcome and defined other outcomes as secondary without correction for multiplicity. This is a common hierarchical strategy and Li et al. [30] suggest that there is no need to correct for multiplicity where secondary outcomes address separate objectives and are exploratory. However, we found a large number of trials reported over 10 secondary clinical outcomes and we believe this lack of multiplicity correction or other strategy becomes less justifiable as the number of secondary outcomes increases. Many trials do tend to overinterpret “statistically significant” results on secondary outcomes, or the reader may be liable to when there is ambiguous interpretation. By increasing the number of secondary outcomes, there is a greater chance of a family-wise type I error if interpreted as confirmatory rather than exploratory [31]. We recommend where possible, trialists limit the number of secondary outcomes to those that are maximally important to stakeholders and to interpret al.l secondary outcomes as exploratory rather than confirmatory unless stated otherwise (in the latter case, multiplicity correction should be considered) More development of, and adherence to, core outcome sets in mental health would help with this issue.

A substantial proportion of trials specified multiple primary outcomes, CONSORT 2010 guidance warns against this because of the “the problems of interpretation associated with multiplicity of analyses” [32]. A small number of these trials did use a multiplicity correction but most did not, as was also found by Vickerstaff et al. previously [10]. This lack of multiplicity correction contradicts regulatory guidance [12–14] and advice on multiplicity [30]. We recommend that trials with multiple primary outcomes use multiplicity correction, another multiple outcome strategy or clearly state outcomes as co-primary i.e., all primary outcomes have to show a significant effect to conclude success.

Anecdotally, a number of trials that had multiple primary outcomes did not explicitly acknowledge that they had used multiple primary outcomes, stating a single primary “outcome” but measuring this outcome with several different measures. Trialists need to be clear that these do in fact constitute multiple primary outcomes, and therefore a strategy to handle these is warranted.

A small minority of trials did not define any clear primary outcome(s). This clearly contradicts CONSORT guidance. This proportion is similar to that found by Vickerstaff et al. [10] suggesting no improvement since then. The latest version of CONSORT guidance was published in 2010, and all journals included in the review state that trials should follow this guidance. Journal editors and peer reviewers need to be more stringent in applying the CONSORT guidance on specifying a clear primary outcome(s).

A large number of trials reported outcomes that were not defined as primary, secondary or exploratory and counting the number of clinical outcomes was often not straightforward because of unclear reporting. We recommend that all outcomes are reported in a clear hierarchy and are linked to clear objectives, to aid interpretation. If outcomes other than those defined as primary/secondary are reported, these should be clearly labelled to indicate how these should be interpreted, for example as exploratory or mechanistic outcomes. For example, the FDA [14] recommends a hierarchy of primary, secondary and exploratory endpoints with primary endpoints those required for marketing approval, secondary endpoints those that support the primary endpoints or demonstrate additional clinical effects and exploratory endpoints that may be helpful for developing new hypotheses.

We did not find that trials of complex interventions reported more outcomes on average than medicinal drug trials; however, a larger percentage did use multiple primary outcomes, highlighting the difficulty in choosing a single primary outcome for complex intervention trials on transdiagnostic populations.

For trials that recorded repeated measures of the primary outcome, most trials appropriately modelled these in a single model, although many trials (> 40%) did not appear to pre-specify a single timepoint as the primary contrast. CONSORT 2010 guidance states “When outcomes are assessed at several time points after randomisation, authors should also indicate the pre-specified time point of primary interest“. Mental health trials need to improve their compliance with this part of the guidance. Although not treated as such in this review, multiple repeated measures of the outcome could be considered separate outcomes and are also prone to problems of interpretation with respect to multiplicity if a single primary time point is not pre-specified [30].

As noted in the results 68% of trials used a multicomponent measure such as a questionnaire which usually include sub-domains and individual items. There measures will often give a single total score; however, if multiple scores are reported from a multicomponent measure as occurred for 15% of trials, (especially where correlated and/or addressing the same hypothesis) we think multiplicity correction, or another strategy is warranted.

### Comparison to other disease areas

The issue of lack of multiplicity correction for multiple outcomes is not unique to mental health trials; a similar review in cardiovascular trials [33] showed that multiplicity adjustments were also infrequently reported where they have multiple primary outcomes (or other multiplicity). A broader survey of CTUs in the UK [34] also showed that adjustment was not always made for multiplicity across a range of pragmatic RCTs.

Other disease areas do appear to use “multiple outcome strategies” more often than we have shown for mental health trials in this review. In particular it is common in cardiovascular trials to use composites [35, 36] (e.g. the win ratio [22]) or joint modelling approaches [37] to combine outcomes such as survival and functioning. We think it is important that these are also considered in mental health trials where the primary outcome(s) of interest are typically not “hard” outcomes such as survival, and as such there is less agreement between clinicians and patients as to which is the important outcome. Therefore, multiple outcomes need to be incorporated in the primary analysis to address the interpretation of benefit to both of these groups of stakeholders, as well as to potentially increase efficiency.

### Limitations

The main limitation of this work is that, for practical reasons, we restricted the search criteria to selected leading journals and specific years published (2019–2020) and so the review is not fully systematic. Our expectation was that this would capture a cross-section at the upper end of current practice in mental health trials, in order to examine these issues in studies which are otherwise likely to be of high methodological quality. However, selecting leading journals does not necessarily make this true. Conversely, if it were true, we may have under-represented the methodological issues in the reporting of multiple outcomes across the totality of published mental health trials. Also consequent to this selection, the review may not be entirely representative, for example we may not have captured the totality of trials across different mental health conditions.

By using a restricted number of years, we could also not examine trends over time other than by comparing to previous reviews.

We only reviewed main papers and not additional supporting material such as appendices and registered protocols. Whilst these may have added additional detail that made reporting clearer, we think that the information we aimed to capture should really be clear from the main paper as most readers will not read this additional material. However, it is possible using the additional material could have demonstrated more outcome reporting issues that are not covered in this review, such as primary outcome switching [38].

As described in the supplementary table S1, 9 of the studies started recruitment prior to 2010 and so would not have had CONSORT 2010 guidelines to adhere to at that time. Earlier CONSORT guidelines existed [39] but did not make recommendations as to multiplicity. However, all were published after 2010 and so could have made changes accordingly.

## Conclusions

While many mental health trials do appear to be following current CONSORT 2010 guidance and the CONSORT 2022 Outcomes extension (albeit this was after when these trials were published) in reporting and analysing the primary outcome(s), a small minority are not and improvements in the analysis and reporting of outcomes are still needed.

We found that most trials in mental health are not taking advantage of multiple outcome strategies for the primary analysis. This may be because of the perceived downsides of strategies such as composite outcomes and the complexity of implementing more advanced analytical strategies. Such strategies could be used to gain efficiency, making trials cheaper and/or quicker to run and aid interpretation where multiple outcomes are of interest to patients and clinicians.

To achieve this, more work is needed to show that (i) multivariate modelling strategies exist that would be of benefit (ii) in which scenarios such strategies would increase efficiency/power and (iii) such strategies are relatively easy to use and implement in mental health trials.

## Electronic supplementary material

Below is the link to the electronic supplementary material.

Supplementary Material 1

## Data Availability

A full dataset and the code used is available on GitHub at https://github.kcl.ac.uk/k0957780/Multiple-outcomes-review.

## Abbreviations

BMJ: British Medical Journal
CONSORT: Consolidated Standards of Reporting Trials
FDA: U.S. Food and Drug Administration
fMRI: Functional magnetic resonance imaging
IQR: Interquartile range
JAMA: Journal of the American Medical Association
MHRA: Medicines and Healthcare products Regulatory Agency
NHS: National Health Service
NICE: The National Institute for Health and Care Excellence
NIHR: National Institute for Health and Care Research
PRISMA: Preferred Reporting Items for Systematic Reviews and Meta-Analyses
RCT: Randomised controlled trial
