# Supplementary material for: The analysis and reporting of multiple outcomes in mental health trials: a methodological systematic review
Source: BMC Med Res Methodol. 2024 Dec 21;24:317. doi: 10.1186/s12874-024-02451-8 (PMC11662570; doi:10.1186/s12874-024-02451-8)
Supplement: Supplementary file 1 — Supplementary Material 1 [file 12874_2024_2451_MOESM1_ESM.docx]

**S1: Trial characteristics**

**Table S1a: Additional characteristics of included trials**

|  | N | Mean | SD | LQ | Median | UQ | Min | Max |
| --- | --- | --- | --- | --- | --- | --- | --- | --- |
| Sample size^1^ | 147 | 441 | 1627 | 100 | 175 | 311 | 18 | 19000 |
| Mean Age of participants | 147 | 36 | 15 | 30.4 | 38.0 | 44.4 | 1.8 | 86 |
| % Female | 147 | .56 | .28 | 0.3 | 0.6 | 0.8 | 0 | 1 |

^1^The trial with 18 participants was an fMRI study, the minimum sample size excluding fMRI studies was 22.

**Table S1b: Country participants recruited from of included trials**

| Country | n | (%) |
| --- | --- | --- |
| Australia | 6 | (4.1) |
| Australia, Germany and the Netherlands | 1 | (0.7) |
| Austria | 1 | (0.7) |
| Brazil | 4 | (2.7) |
| Canada | 8 | (5.4) |
| China | 2 | (1.4) |
| Denmark | 4 | (2.7) |
| France | 1 | (0.7) |
| Germany | 5 | (3.4) |
| Germany and Austria | 1 | (0.7) |
| Haiti | 1 | (0.7) |
| India | 2 | (1.4) |
| Israel | 2 | (1.4) |
| Japan | 1 | (0.7) |
| Kenya and Tanzania | 1 | (0.7) |
| Moldova | 1 | (0.7) |
| Nepal | 2 | (1.4) |
| New Zealand | 1 | (0.7) |
| Nigeria | 1 | (0.7) |
| Nigeria and Ghana | 1 | (0.7) |
| Norway | 2 | (1.4) |
| Norway and Austria | 1 | (0.7) |
| Pakistan | 4 | (2.7) |
| Singapore | 1 | (0.7) |
| Sweden | 3 | (2.0) |
| Switzerland | 2 | (1.4) |
| Thailand | 1 | (0.7) |
| The Netherlands | 12 | (8.2) |
| UK | 15 | (10.2) |
| USA | 51 | (34.7) |
| USA and Australia | 2 | (1.4) |
| USA and Canada | 3 | (2.0) |
| USA and Europe | 2 | (1.4) |
| USA, Canada, Europe | 1 | (0.7) |
| USA, Israel and Canada | 1 | (0.7) |

**Table S1c: Year started and ended recruitment of included trials**

| Start year recruitment |  |  |
| --- | --- | --- |
| 2000 | 1 | (0.8) |
| 2003 | 1 | (0.8) |
| 2004 | 1 | (0.8) |
| 2006 | 1 | (0.8) |
| 2007 | 2 | (1.7) |
| 2008 | 1 | (0.8) |
| 2009 | 2 | (1.7) |
| 2010 | 3 | (2.5) |
| 2011 | 7 | (5.8) |
| 2012 | 15 | (12.4) |
| 2013 | 20 | (16.5) |
| 2014 | 22 | (18.2) |
| 2015 | 22 | (18.2) |
| 2016 | 15 | (12.4) |
| 2017 | 3 | (2.5) |
| 2018 | 4 | (3.3) |
| 2019 | 1 | (0.8) |
| End year follow up |  |  |
| 2006 | 1 | (0.8) |
| 2009 | 1 | (0.8) |
| 2011 | 2 | (1.7) |
| 2012 | 2 | (1.7) |
| 2013 | 2 | (1.7) |
| 2014 | 5 | (4.1) |
| 2015 | 16 | (13.2) |
| 2016 | 34 | (28.1) |
| 2017 | 35 | (28.9) |
| 2018 | 15 | (12.4) |
| 2019 | 7 | (5.8) |
| 2020 | 1 | (0.8) |

**S2: Number of outcomes reported and multiple outcome strategies – overall**

**Table 2a - Number of outcomes**

|  | N | Mean | SD | LQ | Median | UQ | Min | Max | |
| --- | --- | --- | --- | --- | --- | --- | --- | --- | --- |
| **Number of primary outcomes** | 144 | 1.6 | 1.6 | 1.0 | 1.0 | 2.0 | 0 | 13 |  |
| **Number of secondary outcomes** | 144 | 5.3 | 4.9 | 1.0 | 4.0 | 8.0 | 0 | 22 |  |
| **Number of exploratory outcomes** | 144 | .27 | 1.6 | 0.0 | 0.0 | 0.0 | 0 | 17 |  |
| **Number of undefined outcomes** | 144 | 1.6 | 3.4 | 0.0 | 0.0 | 1.0 | 0 | 27 |  |
| **Total number of outcomes** | 144 | 8.8 | 5.5 | 5.0 | 8.0 | 11.0 | 1 | 35 |  |

*Excludes 3 fMRI studies

**Table S2b - Multiple outcome strategy^1^**

| **Was the primary outcome clearly stated or inferable?** |  |  |
| --- | --- | --- |
| Clearly stated | 126 | (87.5) |
| Inferable | 8 | (4.9) |
| Unclear or none stated | 10 | (7.6) |
| **Number of primary outcomes?** |  |  |
| Single primary outcome |  | (70.1) |
| Clearly stated Multiple primary outcomes | 29 | (20.1) |
| Inferred multiple primary outcomes | 4 | (2.8) |
| Unclear | 10 | (6.9) |
| **Was a multiple outcome strategy used for the primary analysis?** |  |  |
| No | 129 | (89.6) |
| Yes | 15 | (10.4) |
| **If so which Multiple outcome strategy used?** |  |  |
| Multiple primaries with multiplicity correction | 6 | (40.0) |
| Percentage composite – unclear weighting | 1 | (6.7) |
| Continuous composite – equal weighting | 1 | (6.7) |
| Continuous composite – unclear weighting | 1 | (6.7) |
| Event composite (with assumed equal weighting) | 5 | (33.3) |
| Unclear | 1 | (6.7) |
| **Was multiplicity correction used for other (e.g. secondary) outcomes?** |  |  |
| No | 119 | (82.6) |
| Yes | 25 | (17.4) |

**^1^**This table excludes the 3 fMRI studies

**S3: Number of outcomes reported and multiple outcome strategies – by Not for profit vs For profit sponsors**

**Table S3a - Number of outcomes by for profit/not for profit^1^**

|  | **Academic or not for profit** | | | | | | | | **Pharmaceutical or for-profit** | | | | | | | | |  |
| --- | --- | --- | --- | --- | --- | --- | --- | --- | --- | --- | --- | --- | --- | --- | --- | --- | --- | --- |
|  | N | Mean | SD | LQ | Median | UQ | Min | Max | | N | Mean | SD | LQ | Median | UQ | Min | Max | |
| **Number of primary outcomes** | 129 | 1.6 | 1.7 | 1.0 | 1.0 | 2.0 | 0 | 13 | | 15 | 1.2 | .56 | 1.0 | 1.0 | 1.0 | 1 | 3 | |
| **Number of secondary outcomes** | 129 | 5.4 | 5.1 | 2.0 | 4.0 | 8.0 | 0 | 22 | | 15 | 4.5 | 3.6 | 1.0 | 4.0 | 8.0 | 0 | 11 | |
| **Number of exploratory outcomes** | 129 | .29 | 1.7 | 0.0 | 0.0 | 0.0 | 0 | 17 | | 15 | .13 | .35 | 0.0 | 0.0 | 0.0 | 0 | 1 | |
| **Number of undefined outcomes** | 129 | 1.5 | 3.5 | 0.0 | 0.0 | 1.0 | 0 | 27 | | 15 | 2.1 | 3 | 0.0 | 0.0 | 5.0 | 0 | 8 | |
| **Total number of outcomes** | 129 | 8.9 | 5.7 | 5.0 | 8.0 | 11.0 | 1 | 35 | | 15 | 7.9 | 3.8 | 5.0 | 7.0 | 11.0 | 2 | 15 | |

**^1^**This table excludes the 3 fMRI studies

**Table S3b - Multiple outcome strategy by profit/not for profit^1^**

|  | **Academic or not for profit** | | **Pharmaceutical or for-profit** | | **Total** | |
| --- | --- | --- | --- | --- | --- | --- |
| **Was the primary outcome clearly stated or inferable?** |  |  |  |  |  |  |
| Clearly stated | 112 | (86.8) | 14 | (93.3) | 126 | (87.5) |
| Inferable | 6 | (4.7) | 1 | (6.7) | 7 | (4.9) |
| Unclear or none stated | 11 | (8.5) | 0 | (0.0) | 11 | (7.6) |
| **Number of primary outcomes?** |  |  |  |  |  |  |
| Single primary outcome | 88 | (68.2) | 13 | (86.7) | 101 | (70.1) |
| Multiple primary outcomes or unclear | 41 | (31.8) | 2 | (13.3) | 43 | (29.9) |
| **Was a multiple outcome strategy used for the primary analysis?** |  |  |  |  |  |  |
| No | 119 | (92.2) | 10 | (66.7) | 129 | (89.6) |
| Yes | 10 | (7.8) | 5 | (33.3) | 15 | (10.4) |
| **If so which Multiple outcome strategy used?** |  |  |  |  |  |  |
| Adjusted multiple primaries | 4 | (40.0) | 2 | (40.0) | 6 | (40.0) |
| Composite percentage – unclear weighting | 0 | (0.0) | 1 | (20.0) | 1 | (6.7) |
| Continuous composite – equal weighting | 1 | (10.0) | 0 | (0.0) | 1 | (6.7) |
| Continuous composite – unclear weighting | 1 | (10.0) | 0 | (0.0) | 1 | (6.7) |
| Event composite (with assumed equal weighting) | 3 | (30.0) | 2 | (40.0) | 5 | (33.3) |
| Unclear | 1 | (10.0) | 0 | (0.0) | 1 | (6.7) |
| **Was multiplicity correction used for other outcomes?** |  |  |  |  |  |  |
| No | 109 | (84.5) | 10 | (66.7) | 119 | (82.6) |
| Yes | 20 | (15.5) | 5 | (33.3) | 25 | (17.4) |

**^1^**This table excludes the 3 fMRI studies

**S4: Number of outcomes reported and multiple outcome strategies – by Complex intervention vs Other (Drug or non-complex intervention)**

**Table S4a - Number of outcomes by drug/complex intervention^1^**

|  | **Drug or non-complex intervention** | | | | | | | | | | | | | | | **Complex Intervention** | | | | | | | | | | | | | | | |  |
| --- | --- | --- | --- | --- | --- | --- | --- | --- | --- | --- | --- | --- | --- | --- | --- | --- | --- | --- | --- | --- | --- | --- | --- | --- | --- | --- | --- | --- | --- | --- | --- | --- |
|  | N | Mean | | SD | | LQ | | Median | | UQ | | Min | | Max | | N | | Mean | | SD | | LQ | | Median | | UQ | | Min | | Max | |  |
| **Number of primary outcomes** | 41 | | 1.3 | | .78 | | 1.0 | | 1.0 | | 1.0 | | 0 | | 5 | | 103 | | 1.7 | | 1.8 | | 1.0 | | 1.0 | | 2.0 | | 0 | | 13 | |
| **Number of secondary outcomes** | 41 | | 5.2 | | 4.3 | | 2.0 | | 5.0 | | 7.0 | | 0 | | 20 | | 103 | | 5.4 | | 5.2 | | 1.0 | | 4.0 | | 8.0 | | 0 | | 22 | |
| **Number of exploratory outcomes** | 41 | | .049 | | .22 | | 0.0 | | 0.0 | | 0.0 | | 0 | | 1 | | 103 | | .36 | | 1.8 | | 0.0 | | 0.0 | | 0.0 | | 0 | | 17 | |
| **Number of undefined outcomes** | 41 | | 2.3 | | 5 | | 0.0 | | 0.0 | | 4.0 | | 0 | | 27 | | 103 | | 1.3 | | 2.5 | | 0.0 | | 0.0 | | 1.0 | | 0 | | 12 | |
| **Total number of outcomes** | 41 | | 8.9 | | 5.8 | | 6.0 | | 8.0 | | 11.0 | | 2 | | 35 | | 103 | | 8.8 | | 5.4 | | 5.0 | | 8.0 | | 11.0 | | 1 | | 24 | |

**Table S4b - Multiple outcome strategy by drug/complex intervention^1^**

|  | **Drug or non-complex intervention** | | **Complex intervention** | | **Total** | |
| --- | --- | --- | --- | --- | --- | --- |
| **Was the primary outcome clearly stated or inferable?** |  |  |  |  |  |  |
| Clearly stated | 36 | (87.8) | 90 | (87.4) | 126 | (87.5) |
| Inferable | 3 | (7.3) | 4 | (3.9) | 7 | (4.9) |
| Unclear or none stated | 2 | (4.9) | 9 | (8.7) | 11 | (7.6) |
| **Number of primary outcomes?** |  |  |  |  |  |  |
| Single primary outcome | 32 | (78.0) | 69 | (67.0) | 101 | (70.1) |
| Multiple primary outcomes or unclear | 41 | (31.8) | 2 | (13.3) | 43 | (29.9) |
| **Was a multiple outcome strategy used for the primary analysis?** |  |  |  |  |  |  |
| No | 119 | (92.2) | 10 | (66.7) | 129 | (89.6) |
| Yes | 10 | (7.8) | 5 | (33.3) | 15 | (10.4) |
| **If so which Multiple outcome strategy used?** |  |  |  |  |  |  |
| Adjusted multiple primaries | 1 | (16.7) | 5 | (55.6) | 6 | (40.0) |
| Composite percentage – unclear weighting | 1 | (16.7) | 0 | (0.0) | 1 | (6.7) |
| Continuous composite – equal weighting | 1 | (16.7) | 0 | (0.0) | 1 | (6.7) |
| Continuous composite – unclear weighting | 0 | (0.0) | 1 | (11.1) | 1 | (6.7) |
| Event composite (with assumed equal weighting) | 2 | (33.3) | 3 | (33.3) | 5 | (33.3) |
| Unclear | 1 | (16.7) | 0 | (0.0) | 1 | (6.7) |
| **Was multiplicity correction used for other outcomes?** |  |  |  |  |  |  |
| No | 32 | (78.0) | 87 | (84.5) | 119 | (82.6) |
| Yes | 9 | (22.0) | 16 | (15.5) | 25 | (17.4) |

**^1^**This table excludes the 3 fMRI studies

**S5: Number of outcomes reported and multiple outcome strategies – by Parallel vs Cluster randomised**

**Table S5a: Number of outcomes reported– by Parallel vs Cluster randomised^1^**

|  | **Cluster** | | | | | | | | **Parallel** | | | | | | | |
| --- | --- | --- | --- | --- | --- | --- | --- | --- | --- | --- | --- | --- | --- | --- | --- | --- |
|  | N | Mean | SD | LQ | Median | UQ | Min | Max | N | Mean | SD | LQ | Median | UQ | Min | Max |
| **Number of primary outcomes** | 16 | 1.4 | 1 | 1.0 | 1.0 | 2.0 | 0 | 4 | 118 | 1.5 | 1.4 | 1.0 | 1.0 | 2.0 | 0 | 9 |
| **Number of secondary outcomes** | 16 | 6.6 | 6.5 | 0.5 | 6.5 | 11.0 | 0 | 21 | 118 | 5.2 | 4.8 | 2.0 | 4.0 | 7.0 | 0 | 22 |
| **Number of exploratory outcomes** | 16 | .31 | 1.3 | 0.0 | 0.0 | 0.0 | 0 | 5 | 118 | .29 | 1.7 | 0.0 | 0.0 | 0.0 | 0 | 17 |
| **Number of undefined outcomes** | 16 | 1.6 | 3.3 | 0.0 | 0.0 | 1.5 | 0 | 12 | 118 | 1.4 | 2.6 | 0.0 | 0.0 | 1.0 | 0 | 11 |
| **Total number of outcomes** | 16 | 9.8 | 7 | 3.0 | 10.0 | 12.5 | 1 | 22 | 118 | 8.5 | 4.8 | 5.0 | 7.5 | 11.0 | 2 | 24 |

**^1^**This table excludes the 3 fMRI studies

**Table S5b: Multiple outcome strategy – by Parallel vs Cluster randomised^1^**

|  | **Cluster** | | **Parallel** | | **Total** | |
| --- | --- | --- | --- | --- | --- | --- |
| **Was the primary outcome clearly stated or inferable?** |  |  |  |  |  |  |
| Clearly stated | 14 | (87.5) | 103 | (87.3) | 117 | (87.3) |
| Inferable | 0 | (0.0) | 6 | (5.1) | 6 | (4.5) |
| Unclear or none stated | 2 | (12.5) | 9 | (7.6) | 11 | (8.2) |
| **Number of primary outcomes?** |  |  |  |  |  |  |
| Single primary outcome | 9 | (56.2) | 84 | (71.2) | 93 | (69.4) |
| Multiple primary outcomes or unclear | 7 | (43.8) | 34 | (28.8) | 41 | (30.6) |
| **Was a multiple outcome strategy used for the primary analysis?** |  |  |  |  |  |  |
| No | 15 | (93.8) | 104 | (88.1) | 119 | (88.8) |
| Yes | 1 | (6.2) | 14 | (11.9) | 15 | (11.2) |
| **If so which Multiple outcome strategy used?** |  |  |  |  |  |  |
| Adjusted multiple primaries | 0 | (0.0) | 6 | (42.9) | 6 | (40.0) |
| Composite percentage – unclear weighting | 0 | (0.0) | 1 | (7.1) | 1 | (6.7) |
| Continuous composite – equal weighting | 0 | (0.0) | 1 | (7.1) | 1 | (6.7) |
| Continuous composite – unclear weighting | 0 | (0.0) | 1 | (7.1) | 1 | (6.7) |
| Event composite (with assumed equal weighting) | 1 | (100.0) | 4 | (28.6) | 5 | (33.3) |
| Unclear | 0 | (0.0) | 1 | (7.1) | 1 | (6.7) |
| **Was multiplicity correction used for other outcomes?** |  |  |  |  |  |  |
| No | 16 | (100.0) | 97 | (82.2) | 113 | (84.3) |
| Yes | 0 | (0.0) | 21 | (17.8) | 21 | (15.7) |

**^1^**This table excludes the 3 fMRI studies

**S6: Analysis and reporting of longitudinal data for primary analysis**

**Table S6a: Analysis and reporting of longitudinal data for primary analysis**

| **Was the primary outcome reported and analysed at multiple timepoints?** |  |  |
| --- | --- | --- |
| No | 28 | (19.0) |
| Yes | 119 | (81.0) |
| **If so, was there a pre-specified single timepoint defined as the primary contrast?** |  |  |
| No | 51 | (42.9) |
| Yes | 68 | (57.1) |
| **Were the multiple timepoints analysed separately or in a single model?** |  |  |
| Both | 4 | (3.4) |
| Single model | 102 | (85.7) |
| Unclear | 6 | (5.0) |
| Separately | 7 | (5.9) |
| **Were treatment effects reported for each individual time point or as a summary measure?** |  |  |
| Individual time points | 50 | (47.2) |
| Summary measure | 28 | (26.4) |
| Both individual timepoints and summary | 27 | (25.5) |
| Unclear | 1 | (0.9) |

**S7. Full search criteria**

Ovid MEDLINE database search criteria:

1. (“Trial” in title or abstract **AND** “Random*” in title or abstract*)* **OR** “Random allocation” in MESH term OR “randomized controlled trial” publication type
2. **Journal =** The British Journal Of Psychiatry **OR** Lancet Psychiatry **OR** JAMA Psychiatry **OR** Journal Of The American Academy Of Child And Adolescent Psychiatry **OR** Psychological Medicine **OR** American Journal Of Psychiatry **OR** Depression and Anxiety
3. (Journal = The Lancet **OR** BMJ **OR** Nature) **AND** ((depress* or behav* or psych*) in multi-purpose fields. **OR** mental disorders/ MESH term)
4. Year Published = 2019 **OR** 2020
5. 1 **AND** (2 **OR** 3) **AND** 4

**S8. References for all included trials**

1.

Andersson E, Ljotsson B, Hedman-Lagerlof M, Nygren L, Persson M, Rosengren K, et al. Targeting excessive worry with internet-based extinction therapy: a randomised controlled trial with mediation analysis and economical evaluation. Psychol Med. 2020;1–11.

2.

Attia E, Steinglass JE, Walsh BT, Wang Y, Wu P, Schreyer C, et al. Olanzapine Versus Placebo in Adult Outpatients With Anorexia Nervosa: A Randomized Clinical Trial. Am J Psychiatry. 2019;176(6):449–56.

3.

Axelsson E, Andersson E, Ljotsson B, Bjorkander D, Hedman-Lagerlof M, Hedman-Lagerlof E. Effect of Internet vs Face-to-Face Cognitive Behavior Therapy for Health Anxiety: A Randomized Noninferiority Clinical Trial. JAMA Psychiatry. 2020;77(9):915–24.

4.

Axinn WG, Chardoul S, Gatny H, Ghimire DJ, Smoller JW, Zhang Y, et al. Using life history calendars to improve measurement of lifetime experience with mental disorders. Psychol Med. 2020;50(3):515–22.

5.

Basanovic J, Grafton B, Ford A, Hirani V, Glance D, MacLeod C, et al. Cognitive bias modification to prevent depression (COPE): results of a randomised controlled trial. Psychol Med. 2020;50(15):2514–25.

6.

Bastiaansen JA, Ornee DA, Meurs M, Oldehinkel AJ. An evaluation of the efficacy of two add-on ecological momentary intervention modules for depression in a pragmatic randomized controlled trial (ZELF-i). Psychol Med. 2020;1–10.

7.

Becker SP, Epstein JN, Tamm L, Tilford AA, Tischner CM, Isaacson PA, et al. Shortened Sleep Duration Causes Sleepiness, Inattention, and Oppositionality in Adolescents With Attention-Deficit/Hyperactivity Disorder: Findings From a Crossover Sleep Restriction/Extension Study. J Am Acad Child Adolesc Psychiatry. 2019;58(4):433–42.

8.

Berk M, Woods RL, Nelson MR, Shah RC, Reid CM, Storey E, et al. Effect of Aspirin vs Placebo on the Prevention of Depression in Older People: A Randomized Clinical Trial. JAMA Psychiatry. 2020;77(10):1012–20.

9.

Best MW, Milanovic M, Iftene F, Bowie CR. A Randomized Controlled Trial of Executive Functioning Training Compared With Perceptual Training for Schizophrenia Spectrum Disorders: Effects on Neurophysiology, Neurocognition, and Functioning. Am J Psychiatry. 2019;176(4):297–306.

10.

Bohus M, Kleindienst N, Hahn C, Muller-Engelmann M, Ludascher P, Steil R, et al. Dialectical Behavior Therapy for Posttraumatic Stress Disorder (DBT-PTSD) Compared With Cognitive Processing Therapy (CPT) in Complex Presentations of PTSD in Women Survivors of Childhood Abuse: A Randomized Clinical Trial. JAMA Psychiatry. 2020;77(12):1235–45.

11.

Boterhoven de Haan KL, Lee CW, Fassbinder E, van Es SM, Menninga S, Meewisse ML, et al. Imagery rescripting and eye movement desensitisation and reprocessing as treatment for adults with post-traumatic stress disorder from childhood trauma: randomised clinical trial. Br J Psychiatry. 2020;217(5):609–15.

12.

Bradley ER, Brustkern J, De Coster L, van den Bos W, McClure SM, Seitz A, et al. Victory is its own reward: oxytocin increases costly competitive behavior in schizophrenia. Psychol Med. 2020;50(4):674–82.

13.

Brenes GA, Divers J, Miller ME, Anderson A, Hargis G, Danhauer SC. Comparison of cognitive-behavioral therapy and yoga for the treatment of late-life worry: A randomized preference trial. Depress Anxiety. 2020;37(12):1194–207.

14.

Brookman-Frazee L, Roesch S, Chlebowski C, Baker-Ericzen M, Ganger W. Effectiveness of Training Therapists to Deliver An Individualized Mental Health Intervention for Children With ASD in Publicly Funded Mental Health Services: A Cluster Randomized Clinical Trial. JAMA Psychiatry. 2019;76(6):574–83.

15.

Brown HE, Freudenreich O, Fan X, Heard SO, Goff D, Petrides G, et al. Efficacy and Tolerability of Adjunctive Intravenous Sodium Nitroprusside Treatment for Outpatients With Schizophrenia: A Randomized Clinical Trial. JAMA Psychiatry. 2019;76(7):691–9.

16.

Bruijniks SJE, Lemmens LHJM, Hollon SD, Peeters FPML, Cuijpers P, Arntz A, et al. The effects of once- versus twice-weekly sessions on psychotherapy outcomes in depressed patients. Br J Psychiatry. 2020;216(4):222–30.

17.

Bryant RA, Kenny L, Rawson N, Cahill C, Joscelyne A, Garber B, et al. Efficacy of exposure-based cognitive behaviour therapy for post-traumatic stress disorder in emergency service personnel: a randomised clinical trial. Psychol Med. 2019;49(9):1565–73.

18.

Burger H, Verbeek T, Aris-Meijer JL, Beijers C, Mol BW, Hollon SD, et al. Effects of psychological treatment of mental health problems in pregnant women to protect their offspring: randomised controlled trial. Br J Psychiatry. 2020;216(4):182–8.

19.

Cardi V, Albano G, Ambwani S, Cao L, Crosby RD, Macdonald P, et al. A randomised clinical trial to evaluate the acceptability and efficacy of an early phase, online, guided augmentation of outpatient care for adults with anorexia nervosa. Psychol Med. 2020;50(15):2610–21.

20.

Carl JR, Miller CB, Henry AL, Davis ML, Stott R, Smits JAJ, et al. Efficacy of digital cognitive behavioral therapy for moderate-to-severe symptoms of generalized anxiety disorder: A randomized controlled trial. Depress Anxiety. 2020;37(12):1168–78.

21.

Carmi L, Tendler A, Bystritsky A, Hollander E, Blumberger DM, Daskalakis J, et al. Efficacy and Safety of Deep Transcranial Magnetic Stimulation for Obsessive-Compulsive Disorder: A Prospective Multicenter Randomized Double-Blind Placebo-Controlled Trial. Am J Psychiatry. 2019;176(11):931–8.

22.

Cassetta BD, Tomfohr-Madsen LM, Goghari VM. A randomized controlled trial of working memory and processing speed training in schizophrenia. Psychol Med. 2019;49(12):2009–19.

23.

Cheng P, Luik AI, Fellman-Couture C, Peterson E, Joseph CLM, Tallent G, et al. Efficacy of digital CBT for insomnia to reduce depression across demographic groups: a randomized trial. Psychol Med. 2019;49(3):491–500.

24.

Christensen TN, Wallstrom IG, Stenager E, Bojesen AB, Gluud C, Nordentoft M, et al. Effects of Individual Placement and Support Supplemented With Cognitive Remediation and Work-Focused Social Skills Training for People With Severe Mental Illness: A Randomized Clinical Trial. JAMA Psychiatry. 2019;76(12):1232–40.

25.

Coffin PO, Santos GM, Hern J, Vittinghoff E, Walker JE, Matheson T, et al. Effects of Mirtazapine for Methamphetamine Use Disorder Among Cisgender Men and Transgender Women Who Have Sex With Men: A Placebo-Controlled Randomized Clinical Trial. JAMA Psychiatry. 2020;77(3):246–55.

26.

Comtois KA, Kerbrat AH, DeCou CR, Atkins DC, Majeres JJ, Baker JC, et al. Effect of Augmenting Standard Care for Military Personnel With Brief Caring Text Messages for Suicide Prevention: A Randomized Clinical Trial. JAMA Psychiatry. 2019;76(5):474–83.

27.

Correll CU, Davis RE, Weingart M, Saillard J, O’Gorman C, Kane JM, et al. Efficacy and Safety of Lumateperone for Treatment of Schizophrenia: A Randomized Clinical Trial. JAMA Psychiatry. 2020;77(4):349–58.

28.

Correll CU, Newcomer JW, Silverman B, DiPetrillo L, Graham C, Jiang Y, et al. Effects of Olanzapine Combined With Samidorphan on Weight Gain in Schizophrenia: A 24-Week Phase 3 Study. Am J Psychiatry. 2020;177(12):1168–78.

29.

Dakwar E, Nunes EV, Hart CL, Foltin RW, Mathew SJ, Carpenter KM, et al. A Single Ketamine Infusion Combined With Mindfulness-Based Behavioral Modification to Treat Cocaine Dependence: A Randomized Clinical Trial. Am J Psychiatry. 2019;176(11):923–30.

30.

Daly EJ, Trivedi MH, Janik A, Li H, Zhang Y, Li X, et al. Efficacy of Esketamine Nasal Spray Plus Oral Antidepressant Treatment for Relapse Prevention in Patients With Treatment-Resistant Depression: A Randomized Clinical Trial. JAMA Psychiatry. 2019;76(9):893–903.

31.

Danielson CK, Adams Z, McCart MR, Chapman JE, Sheidow AJ, Walker J, et al. Safety and Efficacy of Exposure-Based Risk Reduction Through Family Therapy for Co-occurring Substance Use Problems and Posttraumatic Stress Disorder Symptoms Among Adolescents: A Randomized Clinical Trial. JAMA Psychiatry. 2020;77(6):574–86.

32.

Davey CG, Chanen AM, Hetrick SE, Cotton SM, Ratheesh A, Amminger GP, et al. The addition of fluoxetine to cognitive behavioural therapy for youth depression (YoDA-C): a randomised, double-blind, placebo-controlled, multicentre clinical trial. Lancet Psychiatry. 2019;6(9):735–44.

33.

de Almeida Sampaio TP, Jorge RC, Martins DS, Gandarela LM, Hayes-Skelton S, Bernik MA, et al. Efficacy of an acceptance-based group behavioral therapy for generalized anxiety disorder. Depress Anxiety. 2020;37(12):1179–93.

34.

de Jong S, van Donkersgoed RJM, Timmerman ME, Aan Het Rot M, Wunderink L, Arends J, et al. Metacognitive reflection and insight therapy (MERIT) for patients with schizophrenia. Psychol Med. 2019;49(2):303–13.

35.

Dennis CL, Grigoriadis S, Zupancic J, Kiss A, Ravitz P. Telephone-based nurse-delivered interpersonal psychotherapy for postpartum depression: nationwide randomised controlled trial. Br J Psychiatry. 2020;216(4):189–96.

36.

DeRubeis RJ, Zajecka J, Shelton RC, Amsterdam JD, Fawcett J, Xu C, et al. Prevention of Recurrence After Recovery From a Major Depressive Episode With Antidepressant Medication Alone or in Combination With Cognitive Behavioral Therapy: Phase 2 of a 2-Phase Randomized Clinical Trial. JAMA Psychiatry. 2020;77(3):237–45.

37.

Diamond GS, Kobak RR, Krauthamer Ewing ES, Levy SA, Herres JL, Russon JM, et al. A Randomized Controlled Trial: Attachment-Based Family and Nondirective Supportive Treatments for Youth Who Are Suicidal. J Am Acad Child Adolesc Psychiatry. 2019;58(7):721–31.

38.

Dias A, Azariah F, Anderson SJ, Sequeira M, Cohen A, Morse JQ, et al. Effect of a Lay Counselor Intervention on Prevention of Major Depression in Older Adults Living in Low- and Middle-Income Countries: A Randomized Clinical Trial. JAMA Psychiatry. 2019;76(1):13–20.

39.

Dichtel LE, Carpenter LL, Nyer M, Mischoulon D, Kimball A, Deckersbach T, et al. Low-Dose Testosterone Augmentation for Antidepressant-Resistant Major Depressive Disorder in Women: An 8-Week Randomized Placebo-Controlled Study. Am J Psychiatry. 2020;177(10):965–73.

40.

Doi S, Fujiwara T, Isumi A, Mitsuda N. Preventing postpartum depressive symptoms using an educational video on infant crying: A cluster randomized controlled trial. Depress Anxiety. 2020;37(5):449–57.

41.

Donker T, Cornelisz I, van Klaveren C, van Straten A, Carlbring P, Cuijpers P, et al. Effectiveness of Self-guided App-Based Virtual Reality Cognitive Behavior Therapy for Acrophobia: A Randomized Clinical Trial. JAMA Psychiatry. 2019;76(7):682–90.

42.

Dorsey S, Lucid L, Martin P, King KM, O’Donnell K, Murray LK, et al. Effectiveness of Task-Shifted Trauma-Focused Cognitive Behavioral Therapy for Children Who Experienced Parental Death and Posttraumatic Stress in Kenya and Tanzania: A Randomized Clinical Trial. JAMA Psychiatry. 2020;77(5):464–73.

43.

Ducasse D, Dassa D, Courtet P, Brand-Arpon V, Walter A, Guillaume S, et al. Gratitude diary for the management of suicidal inpatients: A randomized controlled trial. Depress Anxiety. 2019;36(5):400–11.

44.

Durgam S, Chen C, Migliore R, Prakash C, Thase ME. Relapse prevention with levomilnacipran ER in adults with major depressive disorder: A multicenter, randomized, double-blind, placebo-controlled study. Depress Anxiety. 2019;36(3):225–34.

45.

Earley W, Burgess MV, Rekeda L, Dickinson R, Szatmari B, Nemeth G, et al. Cariprazine Treatment of Bipolar Depression: A Randomized Double-Blind Placebo-Controlled Phase 3 Study. Am J Psychiatry. 2019;176(6):439–48.

46.

Espie CA, Emsley R, Kyle SD, Gordon C, Drake CL, Siriwardena AN, et al. Effect of Digital Cognitive Behavioral Therapy for Insomnia on Health, Psychological Well-being, and Sleep-Related Quality of Life: A Randomized Clinical Trial. JAMA Psychiatry. 2019;76(1):21–30.

47.

Faurholt-Jepsen M, Frost M, Christensen EM, Bardram JE, Vinberg M, Kessing LV. The effect of smartphone-based monitoring on illness activity in bipolar disorder: the MONARCA II randomized controlled single-blinded trial. Psychol Med. 2020;50(5):838–48.

48.

Felder JN, Epel ES, Neuhaus J, Krystal AD, Prather AA. Efficacy of Digital Cognitive Behavioral Therapy for the Treatment of Insomnia Symptoms Among Pregnant Women: A Randomized Clinical Trial. JAMA Psychiatry. 2020;77(5):484–92.

49.

Findling RL, McNamara NK, Pavuluri M, Frazier JA, Rynn M, Scheffer R, et al. Lithium for the Maintenance Treatment of Bipolar I Disorder: A Double-Blind, Placebo-Controlled Discontinuation Study. J Am Acad Child Adolesc Psychiatry. 2019;58(2):287-296.e4.

50.

Ford T, Hayes R, Byford S, Edwards V, Fletcher M, Logan S, et al. The effectiveness and cost-effectiveness of the Incredible Years R Teacher Classroom Management programme in primary school children: results of the STARS cluster randomised controlled trial. Psychol Med. 2019;49(5):828–42.

51.

Forlenza OV, Radanovic M, Talib LL, Gattaz WF. Clinical and biological effects of long-term lithium treatment in older adults with amnestic mild cognitive impairment: randomised clinical trial. Br J Psychiatry. 2019;215(5):668–74.

52.

Fuhr DC, Weobong B, Lazarus A, Vanobberghen F, Weiss HA, Singla DR, et al. Delivering the Thinking Healthy Programme for perinatal depression through peers: an individually randomised controlled trial in India. Lancet Psychiatry. 2019;6(2):115–27.

53.

Gilbody S, Peckham E, Bailey D, Arundel C, Heron P, Crosland S, et al. Smoking cessation for people with severe mental illness (SCIMITAR+): a pragmatic randomised controlled trial. Lancet Psychiatry. 2019;6(5):379–90.

54.

Ginat-Frolich R, Klein Z, Aderka IM, Shechner T. Reducing avoidance in adults with high spider fear using perceptual discrimination training. Depress Anxiety. 2019;36(9):859–65.

55.

Goldstein LH, Robinson EJ, Mellers JDC, Stone J, Carson A, Reuber M, et al. Cognitive behavioural therapy for adults with dissociative seizures (CODES): a pragmatic, multicentre, randomised controlled trial. Lancet Psychiatry. 2020;7(6):491–505.

56.

Graham AK, Greene CJ, Kwasny MJ, Kaiser SM, Lieponis P, Powell T, et al. Coached Mobile App Platform for the Treatment of Depression and Anxiety Among Primary Care Patients: A Randomized Clinical Trial. JAMA Psychiatry. 2020;77(9):906–14.

57.

Gross D, Belcher HME, Budhathoki C, Ofonedu ME, Dutrow D, Uveges MK, et al. Reducing Preschool Behavior Problems in an Urban Mental Health Clinic: A Pragmatic, Non-Inferiority Trial. J Am Acad Child Adolesc Psychiatry. 2019;58(6):572-581.e1.

58.

Gureje O, Appiah-Poku J, Bello T, Kola L, Araya R, Chisholm D, et al. Effect of collaborative care between traditional and faith healers and primary health-care workers on psychosis outcomes in Nigeria and Ghana (COSIMPO): a cluster randomised controlled trial. Lancet. 2020;396(10251):612–22.

59.

Gureje O, Oladeji BD, Montgomery AA, Araya R, Bello T, Chisholm D, et al. High- versus low-intensity interventions for perinatal depression delivered by non-specialist primary maternal care providers in Nigeria: cluster randomised controlled trial (the EXPONATE trial). Br J Psychiatry. 2019;215(3):528–35.

60.

Haga SM, Drozd F, Lisoy C, Wentzel-Larsen T, Slinning K. Mamma Mia - A randomized controlled trial of an internet-based intervention for perinatal depression. Psychol Med. 2019;49(11):1850–8.

61.

Haight BR, Learned SM, Laffont CM, Fudala PJ, Zhao Y, Garofalo AS, et al. Efficacy and safety of a monthly buprenorphine depot injection for opioid use disorder: a multicentre, randomised, double-blind, placebo-controlled, phase 3 trial. Lancet. 2019;393(10173):778–90.

62.

Haugland BSM, Haaland AT, Baste V, Bjaastad JF, Hoffart A, Rapee RM, et al. Effectiveness of Brief and Standard School-Based Cognitive-Behavioral Interventions for Adolescents With Anxiety: A Randomized Noninferiority Study. J Am Acad Child Adolesc Psychiatry. 2020;59(4):552-564.e2.

63.

Hildebrandt T, Michaeledes A, Mayhew M, Greif R, Sysko R, Toro-Ramos T, et al. Randomized Controlled Trial Comparing Health Coach-Delivered Smartphone-Guided Self-Help With Standard Care for Adults With Binge Eating. Am J Psychiatry. 2020;177(2):134–42.

64.

Holt RIG, Gossage-Worrall R, Hind D, Bradburn MJ, McCrone P, Morris T, et al. Structured lifestyle education for people with schizophrenia, schizoaffective disorder and first-episode psychosis (STEPWISE): randomised controlled trial. Br J Psychiatry. 2019;214(2):63–73.

65.

Hurd YL, Spriggs S, Alishayev J, Winkel G, Gurgov K, Kudrich C, et al. Cannabidiol for the Reduction of Cue-Induced Craving and Anxiety in Drug-Abstinent Individuals With Heroin Use Disorder: A Double-Blind Randomized Placebo-Controlled Trial. Am J Psychiatry. 2019;176(11):911–22.

66.

Husain MI, Chaudhry IB, Khoso AB, Husain MO, Hodsoll J, Ansari MA, et al. Minocycline and celecoxib as adjunctive treatments for bipolar depression: a multicentre, factorial design randomised controlled trial. Lancet Psychiatry. 2020;7(6):515–27.

67.

Hvenegaard M, Moeller SB, Poulsen S, Gondan M, Grafton B, Austin SF, et al. Group rumination-focused cognitive-behavioural therapy (CBT) v. group CBT for depression: phase II trial. Psychol Med. 2020;50(1):11–9.

68.

Ilgen MA, Coughlin LN, Bohnert ASB, Chermack S, Price A, Kim HM, et al. Efficacy of a Psychosocial Pain Management Intervention for Men and Women With Substance Use Disorders and Chronic Pain: A Randomized Clinical Trial. JAMA Psychiatry. 2020;77(12):1225–34.

69.

Ironside M, Browning M, Ansari TL, Harvey CJ, Sekyi-Djan MN, Bishop SJ, et al. Effect of Prefrontal Cortex Stimulation on Regulation of Amygdala Response to Threat in Individuals With Trait Anxiety: A Randomized Clinical Trial. JAMA Psychiatry. 2019;76(1):71–8.

70.

Jager IJ, Vulink NCC, Bergfeld IO, van Loon AJJM, Denys DAJP. Cognitive behavioral therapy for misophonia: A randomized clinical trial. Depress Anxiety. 2020;

71.

James LE, Welton-Mitchell C, Noel JR, James AS. Integrating mental health and disaster preparedness in intervention: a randomized controlled trial with earthquake and flood-affected communities in Haiti. Psychol Med. 2020;50(2):342–52.

72.

Janssen L, Kan CC, Carpentier PJ, Sizoo B, Hepark S, Schellekens MPJ, et al. Mindfulness-based cognitive therapy v. treatment as usual in adults with ADHD: a multicentre, single-blind, randomised controlled trial. Psychol Med. 2019;49(1):55–65.

73.

Johnsen E, Kroken RA, Loberg EM, Rettenbacher M, Joa I, Larsen TK, et al. Amisulpride, aripiprazole, and olanzapine in patients with schizophrenia-spectrum disorders (BeSt InTro): a pragmatic, rater-blind, semi-randomised trial. Lancet Psychiatry. 2020;7(11):945–54.

74.

Jordans MJD, Luitel NP, Garman E, Kohrt BA, Rathod SD, Shrestha P, et al. Effectiveness of psychological treatments for depression and alcohol use disorder delivered by community-based counsellors: two pragmatic randomised controlled trials within primary healthcare in Nepal. Br J Psychiatry. 2019;215(2):485–93.

75.

Kane JM, Schooler NR, Marcy P, Correll CU, Achtyes ED, Gibbons RD, et al. Effect of Long-Acting Injectable Antipsychotics vs Usual Care on Time to First Hospitalization in Early-Phase Schizophrenia: A Randomized Clinical Trial. JAMA Psychiatry. 2020;77(12):1217–24.

76.

Killackey E, Allott K, Jackson HJ, Scutella R, Tseng YP, Borland J, et al. Individual placement and support for vocational recovery in first-episode psychosis: randomised controlled trial. Br J Psychiatry. 2019;214(2):76–82.

77.

Landgren V, Malki K, Bottai M, Arver S, Rahm C. Effect of Gonadotropin-Releasing Hormone Antagonist on Risk of Committing Child Sexual Abuse in Men With Pedophilic Disorder: A Randomized Clinical Trial. JAMA Psychiatry. 2020;77(9):897–905.

78.

Lazarov A, Suarez-Jimenez B, Abend R, Naim R, Shvil E, Helpman L, et al. Bias-contingent attention bias modification and attention control training in treatment of PTSD: a randomized control trial. Psychol Med. 2019;49(14):2432–40.

79.

Lebowitz ER, Marin C, Martino A, Shimshoni Y, Silverman WK. Parent-Based Treatment as Efficacious as Cognitive-Behavioral Therapy for Childhood Anxiety: A Randomized Noninferiority Study of Supportive Parenting for Anxious Childhood Emotions. J Am Acad Child Adolesc Psychiatry. 2020;59(3):362–72.

80.

Lees D, Frampton CM, Merry SN. Efficacy of a Home Visiting Enhancement for High-Risk Families Attending Parent Management Programs: A Randomized Superiority Clinical Trial. JAMA Psychiatry. 2019;76(3):241–8.

81.

Lely JCG, Knipscheer JW, Moerbeek M, Ter Heide FJJ, van den Bout J, Kleber RJ. Randomised controlled trial comparing narrative exposure therapy with present-centred therapy for older patients with post-traumatic stress disorder. Br J Psychiatry. 2019;214(6):369–77.

82.

Lenze EJ, Stevens A, Waring JD, Pham VT, Haddad R, Shimony J, et al. Augmenting Computerized Cognitive Training With Vortioxetine for Age-Related Cognitive Decline: A Randomized Controlled Trial. Am J Psychiatry. 2020;177(6):548–55.

83.

Lewis G, Duffy L, Ades A, Amos R, Araya R, Brabyn S, et al. The clinical effectiveness of sertraline in primary care and the role of depression severity and duration (PANDA): a pragmatic, double-blind, placebo-controlled randomised trial. Lancet Psychiatry. 2019;6(11):903–14.

84.

Livingston G, Barber J, Marston L, Stringer A, Panca M, Hunter R, et al. Clinical and cost-effectiveness of the Managing Agitation and Raising Quality of Life (MARQUE) intervention for agitation in people with dementia in care homes: a single-blind, cluster-randomised controlled trial. Lancet Psychiatry. 2019;6(4):293–304.

85.

Lloyd-Evans B, Osborn D, Marston L, Lamb D, Ambler G, Hunter R, et al. The CORE service improvement programme for mental health crisis resolution teams: results from a cluster-randomised trial. Br J Psychiatry. 2020;216(6):314–22.

86.

Luo X, Law SF, Wang X, Shi J, Zeng W, Ma X, et al. Effectiveness of an Assertive Community Treatment program for people with severe schizophrenia in mainland China - a 12-month randomized controlled trial. Psychol Med. 2019;49(6):969–79.

87.

Lynch TR, Hempel RJ, Whalley B, Byford S, Chamba R, Clarke P, et al. Refractory depression - mechanisms and efficacy of radically open dialectical behaviour therapy (RefraMED): findings of a randomised trial on benefits and harms. Br J Psychiatry. 2020;216(4):204–12.

88.

Maples-Keller JL, Post LM, Price M, Goodnight JM, Burton MS, Yasinski CW, et al. Investigation of optimal dose of early intervention to prevent posttraumatic stress disorder: A multiarm randomized trial of one and three sessions of modified prolonged exposure. Depress Anxiety. 2020;37(5):429–37.

89.

Marsden J, Stillwell G, James K, Shearer J, Byford S, Hellier J, et al. Efficacy and cost-effectiveness of an adjunctive personalised psychosocial intervention in treatment-resistant maintenance opioid agonist therapy: a pragmatic, open-label, randomised controlled trial. Lancet Psychiatry. 2019;6(5):391–402.

90.

Maselko J, Sikander S, Turner EL, Bates LM, Ahmad I, Atif N, et al. Effectiveness of a peer-delivered, psychosocial intervention on maternal depression and child development at 3 years postnatal: a cluster randomised trial in Pakistan. Lancet Psychiatry. 2020;7(9):775–87.

91.

Matthijssen AFM, Dietrich A, Bierens M, Kleine Deters R, van de Loo-Neus GHH, van den Hoofdakker BJ, et al. Continued Benefits of Methylphenidate in ADHD After 2 Years in Clinical Practice: A Randomized Placebo-Controlled Discontinuation Study. Am J Psychiatry. 2019;176(9):754–62.

92.

McCall WV, Benca RM, Rosenquist PB, Youssef NA, McCloud L, Newman JC, et al. Reducing Suicidal Ideation Through Insomnia Treatment (REST-IT): A Randomized Clinical Trial. Am J Psychiatry. 2019;176(11):957–65.

93.

McClintock SM, Martin DM, Lisanby SH, Alonzo A, McDonald WM, Aaronson ST, et al. Neurocognitive effects of transcranial direct current stimulation (tDCS) in unipolar and bipolar depression: Findings from an international randomized controlled trial. Depress Anxiety. 2020;37(3):261–72.

94.

McClure MM, Graff F, Triebwasser J, Perez-Rodriguez M, Rosell DR, Koenigsberg H, et al. Guanfacine Augmentation of a Combined Intervention of Computerized Cognitive Remediation Therapy and Social Skills Training for Schizotypal Personality Disorder. Am J Psychiatry. 2019;176(4):307–14.

95.

McIntyre RS, Subramaniapillai M, Lee Y, Pan Z, Carmona NE, Shekotikhina M, et al. Efficacy of Adjunctive Infliximab vs Placebo in the Treatment of Adults With Bipolar I/II Depression: A Randomized Clinical Trial. JAMA Psychiatry. 2019;76(8):783–90.

96.

Meadows G, Brophy L, Shawyer F, Enticott JC, Fossey E, Thornton CD, et al. REFOCUS-PULSAR recovery-oriented practice training in specialist mental health care: a stepped-wedge cluster randomised controlled trial. Lancet Psychiatry. 2019;6(2):103–14.

97.

Miklowitz DJ, Schneck CD, Walshaw PD, Singh MK, Sullivan AE, Suddath RL, et al. Effects of Family-Focused Therapy vs Enhanced Usual Care for Symptomatic Youths at High Risk for Bipolar Disorder: A Randomized Clinical Trial. JAMA Psychiatry. 2020;77(5):455–63.

98.

Morin CM, Edinger JD, Beaulieu-Bonneau S, Ivers H, Krystal AD, Guay B, et al. Effectiveness of Sequential Psychological and Medication Therapies for Insomnia Disorder: A Randomized Clinical Trial. JAMA Psychiatry. 2020;77(11):1107–15.

99.

Morland LA, Mackintosh MA, Glassman LH, Wells SY, Thorp SR, Rauch SAM, et al. Home-based delivery of variable length prolonged exposure therapy: A comparison of clinical efficacy between service modalities. Depress Anxiety. 2020;37(4):346–55.

100.

Nickerson A, Byrow Y, Pajak R, McMahon T, Bryant RA, Christensen H, et al. ‘Tell Your Story’: a randomized controlled trial of an online intervention to reduce mental health stigma and increase help-seeking in refugee men with posttraumatic stress. Psychol Med. 2020;50(5):781–92.

101.

Niederkrotenthaler T, Till B. Effects of suicide awareness materials on individuals with recent suicidal ideation or attempt: online randomised controlled trial. Br J Psychiatry. 2020;217(6):693–700.

102.

Norman SB, Trim R, Haller M, Davis BC, Myers US, Colvonen PJ, et al. Efficacy of Integrated Exposure Therapy vs Integrated Coping Skills Therapy for Comorbid Posttraumatic Stress Disorder and Alcohol Use Disorder: A Randomized Clinical Trial. JAMA Psychiatry. 2019;76(8):791–9.

103.

Nuechterlein KH, Subotnik KL, Ventura J, Turner LR, Gitlin MJ, Gretchen-Doorly D, et al. Enhancing return to work or school after a first episode of schizophrenia: the UCLA RCT of Individual Placement and Support and Workplace Fundamentals Module training. Psychol Med. 2020;50(1):20–8.

104.

Palhano-Fontes F, Barreto D, Onias H, Andrade KC, Novaes MM, Pessoa JA, et al. Rapid antidepressant effects of the psychedelic ayahuasca in treatment-resistant depression: a randomized placebo-controlled trial. Psychol Med. 2019;49(4):655–63.

105.

Pedersen HF, Agger JL, Frostholm L, Jensen JS, Ornbol E, Fink P, et al. Acceptance and Commitment group Therapy for patients with multiple functional somatic syndromes: a three-armed trial comparing ACT in a brief and extended version with enhanced care. Psychol Med. 2019;49(6):1005–14.

106.

Pellecchia M, Marcus SC, Spaulding C, Seidman M, Xie M, Rump K, et al. Randomized Trial of a Computer-Assisted Intervention for Children With Autism in Schools. J Am Acad Child Adolesc Psychiatry. 2020;59(3):373–80.

107.

Perlis RH, Dowd D, Fava M, Lencz T, Krause DS. Randomized, controlled, participant- and rater-blind trial of pharmacogenomic test-guided treatment versus treatment as usual for major depressive disorder. Depress Anxiety. 2020;37(9):834–41.

108.

Pettit JW, Bechor M, Rey Y, Vasey MW, Abend R, Pine DS, et al. A Randomized Controlled Trial of Attention Bias Modification Treatment in Youth With Treatment-Resistant Anxiety Disorders. J Am Acad Child Adolesc Psychiatry. 2020;59(1):157–65.

109.

Philip NS, Barredo J, Aiken E, Larson V, Jones RN, Shea MT, et al. Theta-Burst Transcranial Magnetic Stimulation for Posttraumatic Stress Disorder. AJP. 2019 Nov;176(11):939–48.

110.

Phillips JL, Norris S, Talbot J, Birmingham M, Hatchard T, Ortiz A, et al. Single, Repeated, and Maintenance Ketamine Infusions for Treatment-Resistant Depression: A Randomized Controlled Trial. Am J Psychiatry. 2019;176(5):401–9.

111.

Popova V, Daly EJ, Trivedi M, Cooper K, Lane R, Lim P, et al. Efficacy and Safety of Flexibly Dosed Esketamine Nasal Spray Combined With a Newly Initiated Oral Antidepressant in Treatment-Resistant Depression: A Randomized Double-Blind Active-Controlled Study. Am J Psychiatry. 2019;176(6):428–38.

112.

Priebe S, Chevalier A, Hamborg T, Golden E, King M, Pistrang N. Effectiveness of a volunteer befriending programme for patients with schizophrenia: randomised controlled trial. Br J Psychiatry. 2020;217(3):477–83.

113.

Rae Olmsted KL, Bartoszek M, Mulvaney S, McLean B, Turabi A, Young R, et al. Effect of Stellate Ganglion Block Treatment on Posttraumatic Stress Disorder Symptoms: A Randomized Clinical Trial. JAMA Psychiatry. 2020;77(2):130–8.

114.

Rahman A, Khan MN, Hamdani SU, Chiumento A, Akhtar P, Nazir H, et al. Effectiveness of a brief group psychological intervention for women in a post-conflict setting in Pakistan: a single-blind, cluster, randomised controlled trial. Lancet. 2019;393(10182):1733–44.

115.

Raine A, Ang RP, Choy O, Hibbeln JR, Ho RMH, Lim CG, et al. Omega-3 (omega-3) and social skills interventions for reactive aggression and childhood externalizing behavior problems: a randomized, stratified, double-blind, placebo-controlled, factorial trial. Psychol Med. 2019;49(2):335–44.

116.

Ramasubbu R, Clark DL, Golding S, Dobson KS, Mackie A, Haffenden A, et al. Long versus short pulse width subcallosal cingulate stimulation for treatment-resistant depression: a randomised, double-blind, crossover trial. Lancet Psychiatry. 2020;7(1):29–40.

117.

Rauch SAM, Kim HM, Powell C, Tuerk PW, Simon NM, Acierno R, et al. Efficacy of Prolonged Exposure Therapy, Sertraline Hydrochloride, and Their Combination Among Combat Veterans With Posttraumatic Stress Disorder: A Randomized Clinical Trial. JAMA Psychiatry. 2019;76(2):117–26.

118.

Roberge P, Provencher MD, Gaboury I, Gosselin P, Vasiliadis HM, Benoit A, et al. Group transdiagnostic cognitive-behavior therapy for anxiety disorders: a pragmatic randomized clinical trial. Psychol Med. 2020;1–11.

119.

Rogers SJ, Estes A, Lord C, Munson J, Rocha M, Winter J, et al. A Multisite Randomized Controlled Two-Phase Trial of the Early Start Denver Model Compared to Treatment as Usual. J Am Acad Child Adolesc Psychiatry. 2019;58(9):853–65.

120.

Rosenblau G, O’Connell G, Heekeren HR, Dziobek I. Neurobiological mechanisms of social cognition treatment in high-functioning adults with autism spectrum disorder. Psychol Med. 2020;50(14):2374–84.

121.

Rosner R, Rimane E, Frick U, Gutermann J, Hagl M, Renneberg B, et al. Effect of Developmentally Adapted Cognitive Processing Therapy for Youth With Symptoms of Posttraumatic Stress Disorder After Childhood Sexual and Physical Abuse: A Randomized Clinical Trial. JAMA Psychiatry. 2019;76(5):484–91.

122.

Rossler W, Kawohl W, Nordt C, Haker H, Rusch N, Hengartner MP. ‘Placement budgets’ for supported employment: impact on employment rates in a multicentre randomised controlled trial. Br J Psychiatry. 2020;216(6):308–13.

123.

Sander LB, Paganini S, Terhorst Y, Schlicker S, Lin J, Spanhel K, et al. Effectiveness of a Guided Web-Based Self-help Intervention to Prevent Depression in Patients With Persistent Back Pain: The PROD-BP Randomized Clinical Trial. JAMA Psychiatry. 2020;77(10):1001–11.

124.

Schroder J, Werkle N, Cludius B, Jelinek L, Moritz S, Westermann S. Unguided Internet-based cognitive-behavioral therapy for obsessive-compulsive disorder: A randomized controlled trial. Depress Anxiety. 2020;37(12):1208–20.

125.

Segal A, Wald I, Pine DS, Halpern P, Bar-Haim Y. Attention control therapy for acute stress disorder: A randomized controlled trial. Depress Anxiety. 2020;37(10):1017–25.

126.

Segal ZV, Dimidjian S, Beck A, Boggs JM, Vanderkruik R, Metcalf CA, et al. Outcomes of Online Mindfulness-Based Cognitive Therapy for Patients With Residual Depressive Symptoms: A Randomized Clinical Trial. JAMA Psychiatry. 2020;77(6):563–73.

127.

Serfaty M, King M, Nazareth I, Moorey S, Aspden T, Mannix K, et al. Effectiveness of cognitive-behavioural therapy for depression in advanced cancer: CanTalk randomised controlled trial. Br J Psychiatry. 2020;216(4):213–21.

128.

Siegel P, Wang Z, Murray L, Campos J, Sims V, Leighton E, et al. Brain-based mediation of non-conscious reduction of phobic avoidance in young women during functional MRI: a randomised controlled experiment. Lancet Psychiatry. 2020;7(11):971–81.

129.

Sikander S, Ahmad I, Atif N, Zaidi A, Vanobberghen F, Weiss HA, et al. Delivering the Thinking Healthy Programme for perinatal depression through volunteer peers: a cluster randomised controlled trial in Pakistan. Lancet Psychiatry. 2019;6(2):128–39.

130.

Smits ML, Feenstra DJ, Eeren HV, Bales DL, Laurenssen EMP, Blankers M, et al. Day hospital versus intensive out-patient mentalisation-based treatment for borderline personality disorder: multicentre randomised clinical trial. Br J Psychiatry. 2020;216(2):79–84.

131.

Strauss GP, Granholm E, Holden JL, Ruiz I, Gold JM, Kelly DL, et al. The effects of combined oxytocin and cognitive behavioral social skills training on social cognition in schizophrenia. Psychol Med. 2019;49(10):1731–9.

132.

Stulz N, Wyder L, Maeck L, Hilpert M, Lerzer H, Zander E, et al. Home treatment for acute mental healthcare: randomised controlled trial. Br J Psychiatry. 2020;216(6):323–30.

133.

Tan S, Zhu X, Fan H, Tan Y, Yang F, Wang Z, et al. Who will benefit from computerized cognitive remediation therapy? Evidence from a multisite randomized controlled study in schizophrenia. Psychol Med. 2020;50(10):1633–43.

134.

Tasca GA, Koszycki D, Brugnera A, Chyurlia L, Hammond N, Francis K, et al. Testing a stepped care model for binge-eating disorder: a two-step randomized controlled trial. Psychol Med. 2019;49(4):598–606.

135.

Towbin K, Vidal-Ribas P, Brotman MA, Pickles A, Miller KV, Kaiser A, et al. A Double-Blind Randomized Placebo-Controlled Trial of Citalopram Adjunctive to Stimulant Medication in Youth With Chronic Severe Irritability. J Am Acad Child Adolesc Psychiatry. 2020;59(3):350–61.

136.

Valiengo L da CL, Goerigk S, Gordon PC, Padberg F, Serpa MH, Koebe S, et al. Efficacy and Safety of Transcranial Direct Current Stimulation for Treating Negative Symptoms in Schizophrenia: A Randomized Clinical Trial. JAMA Psychiatry. 2020;77(2):121–9.

137.

van der Zweerde T, van Straten A, Effting M, Kyle SD, Lancee J. Does online insomnia treatment reduce depressive symptoms? A randomized controlled trial in individuals with both insomnia and depressive symptoms. Psychol Med. 2019;49(3):501–9.

138.

Wang J, Eccles H, Nannarone M, Schmitz N, Patten S, Lashewicz B. Does providing personalized depression risk information lead to increased psychological distress and functional impairment? Results from a mixed-methods randomized controlled trial. Psychol Med. 2020;1–9.

139.

Waxmonsky JG, Pelham WE 3rd, Campa A, Waschbusch DA, Li T, Marshall R, et al. A Randomized Controlled Trial of Interventions for Growth Suppression in Children With Attention-Deficit/Hyperactivity Disorder Treated With Central Nervous System Stimulants. J Am Acad Child Adolesc Psychiatry. 2020;59(12):1330–41.

140.

Weijers J, Ten Kate C, Viechtbauer W, Rampaart LJA, Eurelings EHM, Selten JP. Mentalization-based treatment for psychotic disorder: a rater-blinded, multi-center, randomized controlled trial. Psychol Med. 2020;1–10.

141.

Weiser M, Levi L, Zamora D, Biegon A, SanGiovanni JP, Davidson M, et al. Effect of Adjunctive Estradiol on Schizophrenia Among Women of Childbearing Age: A Randomized Clinical Trial. JAMA Psychiatry. 2019;76(10):1009–17.

142.

White JS, Lowenstein C, Srivirojana N, Jampaklay A, Dow WH. Incentive programmes for smoking cessation: cluster randomized trial in workplaces in Thailand. BMJ. 2020;371(8900488):m3797.

143.

Wilfley DE, Agras WS, Fitzsimmons-Craft EE, Bohon C, Eichen DM, Welch RR, et al. Training Models for Implementing Evidence-Based Psychological Treatment: A Cluster-Randomized Trial in College Counseling Centers. JAMA Psychiatry. 2020;77(2):139–47.

144.

Wilhelm S, Phillips KA, Greenberg JL, O’Keefe SM, Hoeppner SS, Keshaviah A, et al. Efficacy and Posttreatment Effects of Therapist-Delivered Cognitive Behavioral Therapy vs Supportive Psychotherapy for Adults With Body Dysmorphic Disorder: A Randomized Clinical Trial. JAMA Psychiatry. 2019;76(4):363–73.

145.

Wolfling K, Muller KW, Dreier M, Ruckes C, Deuster O, Batra A, et al. Efficacy of Short-term Treatment of Internet and Computer Game Addiction: A Randomized Clinical Trial. JAMA Psychiatry. 2019;76(10):1018–25.

146.

Wood JJ, Kendall PC, Wood KS, Kerns CM, Seltzer M, Small BJ, et al. Cognitive Behavioral Treatments for Anxiety in Children With Autism Spectrum Disorder: A Randomized Clinical Trial. JAMA Psychiatry. 2020;77(5):474–83.

147.

Zoellner LA, Roy-Byrne PP, Mavissakalian M, Feeny NC. Doubly Randomized Preference Trial of Prolonged Exposure Versus Sertraline for Treatment of PTSD. Am J Psychiatry. 2019;176(4):287–96.
